# The impact of industrial collaborative agglomeration on total factor carbon emission efficiency in China

**DOI:** 10.1038/s41598-023-39631-3

**Published:** 2023-07-31

**Authors:** Honglin Yuan, Jia Liu, Xiaona Li, Shen Zhong

**Affiliations:** 1 Jiangxi University of Finance and Economics, Nanchang, 330000 Jiangxi People’s Republic of China; 2 Harbin University of Commerce, Harbin, 150028 Heilongjiang People’s Republic of China

**Keywords:** Climate sciences, Ecology, Environmental sciences, Environmental social sciences

## Abstract

Improving total factor carbon emission efficiency (TCE) is the key to achieving carbon emission reduction targets while ensuring economic growth. In this paper, the global Malmquist index based on the SBM model is used to measure TCE of 283 cities in China from 2011 to 2019. On this basis, this paper uses the spatial econometric model and intermediary effect model to empirically analyze the impact of industrial co-agglomeration on TCE and its transmission mechanism. Furthermore, considering the differences in geographical location and resource endowment among regions, this paper analyzes the heterogeneous effect of industrial collaboration agglomeration on TCE in different regions and cities. The results show that: (1) Industrial co-agglomeration can improve TCE, and its main transmission channel is technological innovation. (2) Industrial co-agglomeration has a positive spatial spillover effect. Industrial co-agglomeration in one region can improve the TCE in the surrounding regions. (3) Industrial co-agglomeration of cities with different geographic locations and resource endowments has a heterogeneous effect on TCE. Regarding geographical heterogeneity, the industrial co-agglomeration in the eastern region has the greatest promoting effect on TCE, followed by the central region. However, the impact of industrial co-agglomeration in the western region on TCE is not significant. Regarding resource endowment heterogeneity, the industrial co-agglomeration in non-resource-based cities has a greater promoting effect on TCE than that in resource-based cities.

## Introduction

The massive CO_2_ emissions have enhanced the global warming trend, seriously affecting human production and life^1^. In this context, countries around the world are actively exploring how to achieve carbon reduction goals^2^. As the world’s largest energy consumer and CO_2_ emitter^3^, the realization of China’s carbon emission reduction target is crucial for solving the problem of global warming. In 2016, the Chinese government proposed in the Paris Agreement to achieve the carbon peak by 2023 and reduce the carbon emission intensity by 60–65% compared with 2005, showing the determination to deal with the carbon emission problem to the world^4^. As the world’s largest developing country, China’s carbon emission reduction target cannot be achieved at the expense of economy^5^. Therefore, how to alleviate the contradiction between economic growth and CO_2_ emission is an urgent problem for China^6^. Improving TCE is an effective way to solve this problem^7^.

To improve TCE, scholars have discussed the impact of financial development^8^, urbanization level^9^, industrial structure^10^, energy structure^11^, technological progress^12^, transport infrastructure^13^ and other factors on TCE. Among them, industrial co-agglomeration is the main driving force for TCE. Industrial co-agglomeration reduces product transaction costs and increases economies of scale^14^, which allows enterprises to have more funds for low-carbon technology research and development. Meanwhile, industrial co-agglomeration can generate positive knowledge and technology spillover effects^15^, promoting low-carbon development in surrounding areas. However, few studies have conducted theoretical analysis and empirical research on the relationship between industrial co-agglomeration and TCE.

This article theoretically analyzes the impact of industrial co-agglomeration on TCE and its transmission mechanism. On this basis, this paper uses the spatial econometric model and intermediary effect model to empirically test the impact of industrial co-agglomeration on TCE. Considering that each city has different geographical locations and resource endowments, this paper explores the heterogeneous effect of industrial co-agglomeration in different regions and cities with different resource endowments on TCE.

**Figure 1:**
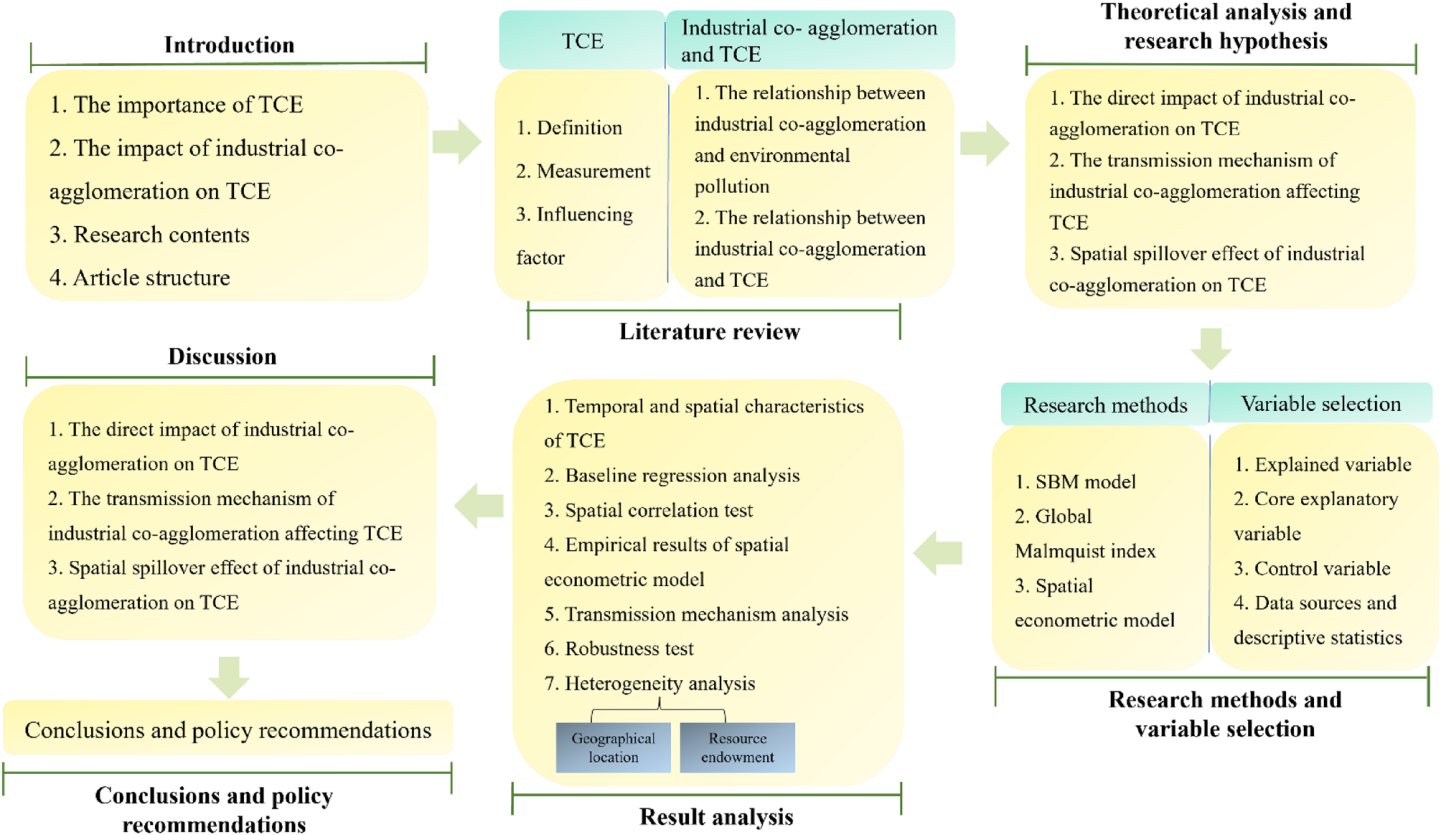
Analysis framework.

The research structure is shown in Fig. 1. Among them, the first part is the introduction. The second part is the literature review. The third part is the theoretical analysis and research hypothesis. The fourth part is the research methods and selection of variables. The fifth part is the result analysis. The sixth part is the discussion. The seventh part is the conclusion and policy recommendations.

## Literature review

With the increasing attention paid to carbon emission reduction, massive scholars studied the efficiency of carbon emission. Carbon emission efficiency mainly includes single-factor carbon emission efficiency (SCE) and TCE^16^. The SCE is defined as CO_2_ emissions per unit of GDP^17,18^, CO_2_ emissions per unit of energy consumption^19^, GDP per unit of CO_2_ emission^20,21^. SCE has the characteristics of simple calculation process and easy understanding. However, it only considers the impact of output on SCE, ignoring the impact of input factors such as capital, labor and energy on SCE^22^. Under the framework of total factor analysis, scholars put forward the concept of TCE. TCE refers to the combination of optimal economic output and CO_2_ emission that can be achieved under the condition that capital, labor and energy input remain unchanged^23^. Compared with SCE, TCE not only considers the CO_2_ emission process, but also considers the influence of various input factors^24^. Scientifically measuring TCE is the premise of exploring how to improve TCE. Measurement methods mainly include stochastic frontier analysis (SFA)^25,26^ and data envelopment analysis (DEA)^27^. However, the SFA needs to take a specific form, which makes it have certain limitations in the measurement of TCE. Since there is no need for model setting and parameter estimation, DEA, such as the traditional DEA^28,29^, SBM^30^ and DDF^31,32^ has been widely used to measure TCE. However, these models can only carry out static analysis of the efficiency of decision units. Currently, the Malmquist index has gradually been used to measure TCE^33,34^. The global Malmquist index is an extension of the Malmquist index. It has the characteristics of transitivity and cyclic accumulation. It can accurately reflect the dynamic change of TCE^35^. However, few scholars currently use this model to measure TCE.

Exploring the impact of various factors on TCE is also a key focus of scholars. It is found that income inequality^36^, urbanization level^37^, industrial structure^38^, digital finance^39^, environmental regulation^40^ and other economic and social factors have different impacts on TCE. Specifically, the optimization of industrial structure and environmental regulation have a positive impact on TCE. The increase of income inequality and the development of digital finance have a negative impact on TCE. The relationship between urbanization level and TCE is inverted U-shaped. In addition to economic and social factors, the introduction of some policies can also have an impact on TCE, such as low-carbon city pilot policy^41,42^ and carbon emission trading policy^43^. Among many influencing factors, the impact of industrial co-agglomeration on TCE cannot be ignored. However, scholars only have analyzed the impact of industrial co-agglomeration on industrial pollutant emission^44^, haze pollution^45^, environmental efficiency^46^ and green development efficiency^15^. The effect of industrial co-agglomeration on TCE is rarely discussed. Li et al.^47^ and Meng et al.^48^ used provincial panel data to empirically analyze the impact of industrial co-agglomeration on CO_2_ emissions, but did not deeply explore its mechanism. Meanwhile, provincial data fails to show differences between regions. Existing studies have found that different cities have different resource endowments, which makes the potential improvement of TCE of cities significantly different^49^. According to the resource curse hypothesis, cities with rich natural resources may be at a disadvantage in carbon emission reduction^50,51^.

In order to make up for the shortcomings of existing literature, this paper makes the following contributions: Firstly, in terms of research methods, this paper incorporates the global Malmquist index based on SBM model and the spatial econometric model into the same framework, which not only makes the measurement of TCE and empirical results more accurate, but also enriches the application of two models in the field of carbon emissions. At present, some scholars use the global Malmquist index based on SBM model to measure TCE^52,53^. Some scholars use spatial econometric models to examine the relationship between industrial agglomeration and carbon emissions^54,55^. Few scholars incorporate them into the same framework. Secondly, in terms of theory, this paper analyzes the impact of industrial co-agglomeration on TCE, and further discusses the mediating role of technological innovation between them. This not only helps us to fully understand the logical relationship between industrial co-agglomeration and TCE, but also enriches the researches on industrial co-agglomeration and TCE. Thirdly, in terms of sample selection, panel data of 283 Chinese cities from 2011 to 2019 is selected as research samples, which fully reflects the regional differences in economic development, industrial structure and resource endowment. Moreover, considering the differences in resource endowments of different cities, this paper analyzes the heterogeneous effects of industrial co-agglomeration on TCE of resource-based cities and non-resource-based cities.

## Theoretical analysis and research hypothesis

Industrial co-agglomeration refers to the aggregation of manufacturing and producer services in a specific area under the influence of output and technology correlation. It is an extension of industrial agglomeration. Its essence still belongs to the research category of industrial agglomeration. The theories related to industrial agglomeration mainly include MAR externality theory, Jacobs externality theory and Porter externality theory. MAR externality theory holds that the agglomeration of similar industries promotes the specialization of labor market and exchange of technology between enterprises, thus exerting the positive externalities of economy^56^. Different from MAR externality theory, Jacobs externality theory believes that the agglomeration of different industries produces complementary effects of capital, technology and labor, thus exerting positive economic externalities^57^. The agglomeration of different industries also has a competitive effect, accelerating the dissemination and absorption of technology between different industries, resulting in greater externalities. Based on the above two theories, Porter (1990) proposed Porter’s externality theory^58^. This theory emphasizes the market competition effect of industrial agglomeration. Benign competition among enterprises in the agglomeration area reduces transaction costs, thus promoting the positive externalities of economy. Industrial co-agglomeration can produce positive economic externalities, thus affecting TCE. This paper analyzes the direct influence, transmission mechanism and spatial spillover effect of industrial co-agglomeration on TCE.

### The direct impact of industrial co-agglomeration on TCE

**Figure 2:**
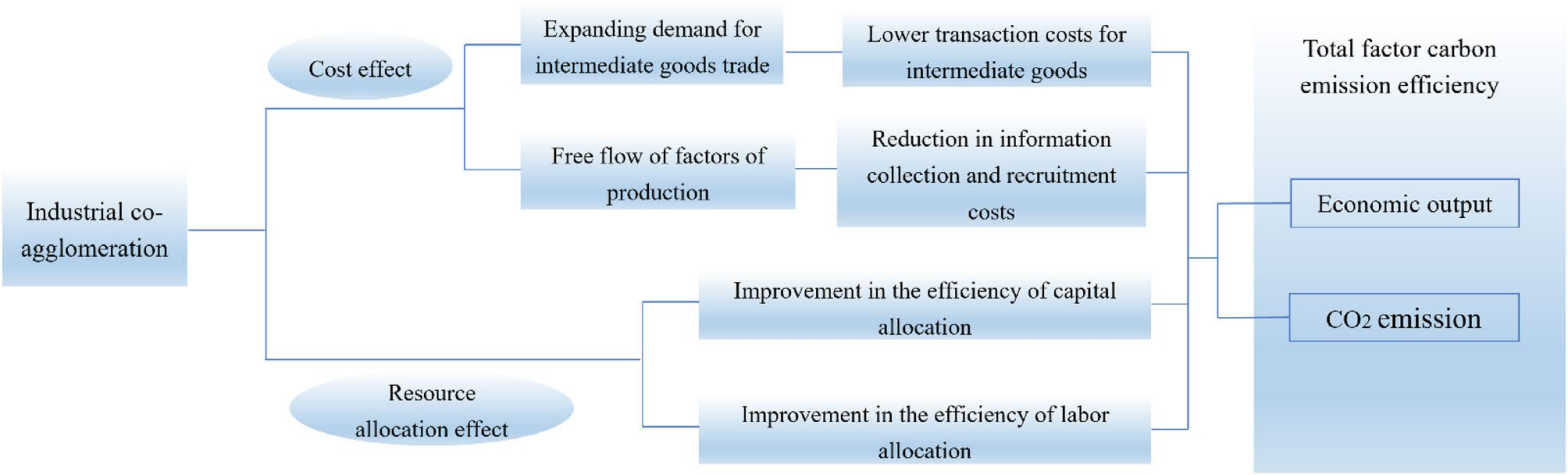
The direct impact of industrial co-agglomeration on TCE.

Industrial co-agglomeration affects TCE mainly through cost effect and resource allocation effect (Fig. 2). Regarding cost effect, industrial co-agglomeration can save enterprise cost not only in the trade of intermediate goods, but also in the flow of production factors. As for intermediate goods trade, due to the refinement of industrial division of labor, enterprises in demand and supply of intermediate goods based on input–output relationship will appear in the collaborative agglomeration area, which reduces the transaction cost of intermediate goods^59^. As for the flow of production factors, industrial co-agglomeration can attract lots of highly skilled talents, which reduces the cost of information collection and talent recruitment. The reduction of product transaction costs, information collection costs and talent recruitment costs can not only increase economic output, but also help enterprises to spend more money on research and development of clean technologies and reduce CO_2_ emissions. Regarding resource allocation effect, industrial co-agglomeration can promote industrial specialization and save transaction costs and information collection costs, which makes enterprises spend more money on the production of environmental protection products. The rational allocation of capital is realized. Meanwhile, the area of industrial co-agglomeration can attract talents from surrounding areas to gather in this area, providing employment opportunities for the labor force in surrounding areas^60^. The allocative efficiency of labor is improved. The improvement of capital and labor allocation efficiency can increase the economic output of enterprises and reduce CO_2_ emissions, thus improving TCE. Hypothesis 1 is proposed.

Hypothesis 1: Industrial co-agglomeration can improve TCE.

### The transmission mechanism of industrial co-agglomeration affecting TCE

**Figure 3:**
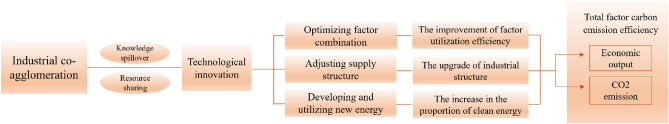
The transmission mechanism of industrial co-agglomeration affecting TCE.

Industrial co-agglomeration has a positive impact on technological innovation through knowledge spillover and resource sharing (Fig. 3). About knowledge spillover, with the expansion of industrial co-agglomeration, the scale of technology and knowledge-intensive producer services gradually expands, which increases the concentration of production factors such as technology and knowledge. Enterprises in the agglomeration area can exchange knowledge and technology, which is conducive to technological innovation in production and emission reduction^61^. China attaches great importance to carbon emissions. In 2009, China put forward the carbon emission reduction target for the first time: to achieve CO_2_ emissions per unit of GDP in 2020 decreased by 40–50% compared with 2005. Since then, a series of environmental protection policies have been introduced. The enforcement of law enforcement is increasing, which makes the technological innovation of enterprises gradually develop towards emission reduction^62^. The technological innovation of enterprises can produce more low-carbon technologies^63^, such as clean energy technologies in front-end production, energy-saving technologies in production processes, and carbon recycling technologies in end-of-pipe treatment, thereby reducing CO_2_ emissions. About resource sharing, industrial co-agglomeration has the characteristics of geographical proximity, industrial association and specialized division of labor. These characteristics create a resource sharing environment for enterprises in the agglomeration area. Specifically speaking, industrial co-agglomeration attracts lots of high-end technical talents and improves the efficiency of labor resource sharing, which provides talent support for technological innovation and R&D. Meanwhile, industrial co-agglomeration has the characteristics of spatial geographical proximity and industrial linkage, which improves the sharing efficiency of resources such as education and facilities^64^ and promotes technological innovation.

Technological innovation can affect TCE by optimizing factor combination, adjusting supply structure and developing and utilizing new energy (Fig. 3). Firstly, technological innovation can affect the input–output model of production factors, optimize the combination of factors, and improve the utilization efficiency of factors. Secondly, with the continuous innovation of technology, the update frequency of products and services is accelerated. The industrial structure is constantly upgrading^65^. Energy conservation and environmental protection industries are growing, which makes TCE improved. Finally, technological innovation reduces the cost of using clean energy such as wind energy and solar energy, which promotes the use of clean energy^66^. The use of clean energy can achieve cleaner production in front-end production^67^, enabling enterprises to reduce carbon dioxide emissions while obtaining economic output. Hypothesis 2 is proposed.

Hypothesis 2: Industrial co-agglomeration can improve TCE by promoting technological innovation.

### Spatial spillover effect of industrial co-agglomeration on TCE

Industrial co-agglomeration has a spatial spillover effect, which is mainly reflected in three aspects: Technological progress, learn effect and collaboration effect. Regarding technological progress, new knowledge and technology can not only spread within the agglomeration industry, but also spread to the surrounding areas^68^. The reason is that the flow and diffusion of knowledge and technology in the surrounding areas do not require high costs. Meanwhile, it can bring higher economic benefits. Regarding the learning effect, the TCE of neighboring regions may be affected by the economic behavior of the region. Under the pressure of political promotion, the surrounding backward areas actively learn the technology and management experience of advanced areas^69^, thus promoting the improvement of TCE. Regarding the collaborative effect, industrial co-agglomeration can promote regional specialized production. With the advancement of regional specialized production, resource-intensive and labor-intensive industries are gradually transferred to regions with low productivity levels. Each region conducts specialized production on the basis of its original comparative advantages, so as to jointly achieve the improvement of TCE. Hypothesis 3 is proposed.

Hypothesis 3: Industrial co-agglomeration has a positive spatial spillover effect on TCE.

## Research methods and variable selection

### Research methods

#### SBM model

Tone^70^ proposed the SBM model considering undesirable outputs. This model cannot only solve the problem of input–output slack, but also solve the problem of efficiency analysis in the presence of undesirable outputs. The specific formula is as follows:

$$\min \rho = \frac{1 - \frac{1}{m}\sum\limits_{i = 1}^{m} \frac{{s_i }^{-} }{x_{ik}}} {1 - \frac{1}{q_1 + q_2}\left( \sum\limits_{r = 1}^{q_1} \frac{s_{r}^{g}} {y_{rk}^{g}} + \sum\limits_{r = 1}^{q_2} \frac{s_{r}^{b}} {y_{rk}^{b}} \right)}$$

1 $$\begin{aligned} s.t. \, \;\; & X\lambda + s - = xk \\ & Yg\lambda - sg = ykg \\ & Yb\lambda - sb = ykb \\ \, & \lambda , \, s - , \, sg, \, sb \ge 0 \\ \end{aligned}$$

Among them, $s = (s - ,\;sb,\;s + )$ is the slack amount of input, expected output and undesirable output; $\rho$ is the efficiency value of decision making unit (DMU), which is between 0 and 1. When $\rho = 1$, $s - = sb = s + = 0$, the DMU is effective. Otherwise, the DMU is invalid.

#### Global Malmquist index

This paper uses the global Malmquist index to measure the TCE. The model can not only analyze the long-term trend of production efficiency, but also avoid the defect of linear programming without solution. According to Oh^71^, the global Malmquist index is defined as follows:

2 $$GML^{t,t + 1} (x^{t} ,\;y^{t} ,\;b^{t} ,\;x^{t + 1} ,\;y^{t + 1} ,\;b^{t + 1} ) = \left\{ \begin{gathered} \frac{{1 + \overrightarrow {{D_{C}^{T} }} (x^{t} ,\;y^{t} ,\;b^{t} ;\;g^{t} )}}{{1 + \overrightarrow {{D_{C}^{T} }} (x^{t + 1} ,\;y^{t + 1} ,\;b^{t + 1} ;\;g^{t + 1} )}} \hfill \\ \times \frac{{1 + \overrightarrow {{D_{C}^{T + 1} }} (x^{t} ,\;y^{t} ,\;b^{t} ;\;g^{t} )}}{{1 + \overrightarrow {{D_{C}^{T + 1} }} (x^{t + 1} ,\;y^{t + 1} ,\;b^{t + 1} ;\;g^{t + 1} )}} \hfill \\ \end{gathered} \right\}^{\frac{1}{2}}$$

Among them, $GML^{t,t + 1}$ is the global Malmquist index from *t* period to *t* + *1* period. $GML^{t,t + 1} > 1$ indicates that the TCE of DMU is improved from *t* period to *t* + *1* period. $GML^{t,t + 1} < 1$ indicates that the TCE of DMU decreases from *t* period to *t* + *1* period.

#### Spatial econometric model

The first law of geography points out that there is a correlation between all things. The closer the distance, the stronger the correlation of things^72^. With the continuous improvement of transportation infrastructure, the communication between regions is deepening and the spatial connection is increasing. The impact of industrial co-agglomeration on TCE may have a spatial correlation. Ignoring spatial correlation may result in inaccurate estimation results. Therefore, spatial econometric models are used to further analyze the impact of industrial co-agglomeration on TCE. Spatial econometric models mainly include spatial autoregressive model (SAR), spatial error model (SEM) and spatial Durbin model (SDM). The formulas are as follows:

SAR:

3 $$\begin{aligned} TCE_{it} & = \alpha_{0} \sum\limits_{j} {W_{ij} } TCE_{it} + \alpha_{1} INA_{it} + \alpha_{2} \ln GDP_{it} + \alpha_{3} GOV_{it} \\ & \quad + \alpha_{4} ROAD_{it} + \alpha_{5} OPEN_{it} + \alpha_{6} HUM_{it} + \mu_{i} + \lambda_{t} + \varepsilon_{it} \\ \end{aligned}$$

SEM:

4 $$\begin{aligned} TCE_{it} & = \beta_{1} INA_{it} + \beta_{2} \ln GDP_{it} + \beta_{3} GOV_{it} + \beta_{4} ROAD_{it} \\ & \quad + \beta_{5} OPEN_{it} + \beta_{6} HUM_{it} + \mu_{i} + \lambda_{t} + \varepsilon_{it} \\ \, & \quad \varepsilon_{it} = \eta \sum\limits_{j} {W_{ij} v_{jt} } \\ \end{aligned}$$

SDM:

5 $$\begin{aligned} TCE_{it} & = \theta_{0} \sum\limits_{j} {W_{ij} } TCE_{it} + \theta_{1} INA_{it} + \theta_{2} \ln GDP_{it} + \theta_{3} GOV_{it} + \theta_{4} ROAD_{it} \\ & \quad + \theta_{5} OPEN_{it} + \theta_{6} HUM_{it} + \rho_{0} \sum\limits_{j} {W_{ij} } INA_{it} + \rho_{1} \sum\limits_{j} {W_{ij} } \ln GDP_{it} \\ & \quad + \rho_{2} \sum\limits_{j} {W_{ij} } GOV_{it} + \rho_{3} \sum\limits_{j} {W_{ij} } ROAD_{it} + \rho_{4} \sum\limits_{j} {W_{ij} } OPEN_{it} \\ & \quad + \rho_{5} \sum\limits_{j} {W_{ij} } HUM_{it} + \mu_{i} + \lambda_{t} + \varepsilon_{it} \\ \end{aligned}$$

Among them, *i* and *t* are region and year, respectively. *TCE* is the total factor carbon emission efficiency of the explained variable. *INA* is the core explanatory variable of industrial co-agglomeration. *lnGDP*, *GOV*, *ROAD*, *OPEN* and *HUM* are the control variables of economic development, government intervention, transportation infrastructure level, opening degree and human capital respectively. $\mu_{i}$ is regional effect; $\lambda_{t}$ is time effect. $\varepsilon_{it}$ is random error term. $W_{ij}$ is spatial weight matrix. Considering the influence of geographical factors on the correlation between variables, this paper selects the geographical distance weight matrix as the spatial weight matrix. The formula is:

6 $$W_{ij} = \left\{ {\begin{array}{*{20}c} {1/d_{ij} } & {\quad \, if\; \, i \ne j} \\ {0 \, } & {\quad \, if\; \, i = j} \\ \end{array} } \right. \,$$

Among them, $d_{ij}$ is the geographical distance between region *i* and region *j*.

### Variable selection

#### Explained variable

The explained variable is TCE. The estimation methods mainly include SFA and DEA. When there are too many input indicators, the SFA cannot accurately calculate the efficiency value. Therefore, this paper selects the global Malmquist index based on SBM model to measure TCE. According to relevant research^73^, this paper selects the following evaluation indicators of TCE (Table 1).

**Table 1:** Input and output index.

Index	Variable	Calculation	Mean	Std. dev.	Min	Max
Input	Stock of capital	$K_{i,t} = K_{i,t - 1} + (1 - \delta )I_{i,t}$	9522.55	10,426.5	42.66	100,732.9
	Labor force	Number of employees	56.71	84.99	4.53	986.87
	Energy consumption	$E_{i,t} = GDP_{i,t} \times EI_{i,t}$	1426.47	1592.02	27.55	11,859
Expected output	GDP	Reduction method	1358.94	1921.89	18.82	18,643.86
Unexpected output	CO_2_ emissions	CEADs database	37.27	40.29	1.34	457.757

Regarding the input indicators, this paper selects the fixed capital stock as the capital input. According to Wang et al.^74^, the perpetual inventory method is used to calculate the fixed capital stock. The specific formula is as follows:

7 $$K_{i,t} = K_{i,t - 1} + (1 - \delta )I_{i,t}$$

Among them, *K*_*i,t*_ is the actual fixed capital stock of city *i* in year *t*. $\delta$ is the capital depreciation rate, which is set to 9.6%. *I*_*i,t*_ is the total fixed asset formation of city *i* in year *t*. Taking 2009 as the base period, this paper uses the fixed asset investment price index to deflate and convert it into the actual value represented by the constant price in 2009.

The number of employees is selected as labor input. The total energy consumption is selected as energy input. Referring to the method of Dhakal^75^, this paper calculates the total energy consumption. The specific formula is as follows:

8 $$E_{i,t} = GDP_{i,t} \times EI_{i,t}$$

Among them, *E*_*i,t*_ is the total energy consumption of city *i* in year *t*. *GDP*_*i,t*_ is the regional GDP of city *i* in year *t*. *EI*_*i,t*_ is the energy intensity of city *i* in year *t*. Assuming that the energy intensity of each city is the same as that of the province.

Regarding the output indicator, the regional GDP is selected as the expected output. Taking 2009 as the base period, this paper uses the GDP deflator to reduce nominal GDP to real GDP at a comparable price. The CO_2_ emissions is selected as undesired outputs. The data comes from the CEADs database.

#### Core explanatory variables

Industrial co-agglomeration is the core explanatory variable. Referring to Ellison et al.^76^, this paper calculates the level of industrial co-agglomeration. The formula is as follows:

9 $$INA_{i} = \left( {1 - \frac{{\left| {LQ_{im} - LQ_{is} } \right|}}{{LQ_{im} + LQ_{is} }}} \right) + (LQ_{im} + LQ_{is} )$$

Among them, *m* is the manufacturing industry. *s* is producer services, including transportation, finance, leasing and business services, information services, science and technology services. *LQ*_*im*_ is the location entropy of manufacturing industry in city *i*. *LQ*_*is*_ is the location entropy of producer services in city *i*. *INA*_*i*_ is the level of co-agglomeration of manufacturing and producer services in city *i*.

#### Control variables

The control variables mainly include economic development, urbanization, government intervention, transportation infrastructure level, opening degree and human capital.

1. Economic development (lnGDP): Rapid economic growth destroys the ecological environment. With the continuous improvement of economic development, enterprises have begun to control pollution and reduce CO_2_ emissions by developing clean technologies^77^. Meanwhile, the government implements more stringent environmental protection measures to improve TCE. The GDP per capita is selected as a measurement index.
2. Government intervention (GOV): Government intervention in the factor market makes production factors occupied by low value-added sectors, reducing production efficiency. Meanwhile, the government invests more money in economic-related fields to develop economy, crowding out investment in the environmental field^78^ and increasing CO_2_ emissions. The reduction of production efficiency and increase of CO_2_ emissions can reduce TCE. The ratio of government fiscal expenditure to GDP is used as a measurement index.
3. Transportation infrastructure level (ROAD): The construction of transportation infrastructure consumes fossil energy and produces large amounts of CO_2_^79^. Meanwhiles, the improvement of transportation infrastructure has a scale economy effect on economic and social factors^13^, which indirectly affects TCE. Per capita road area is used as a measurement index.
4. Openness degree (OPEN): The development of a country’s economy is inseparable from trade with other countries^80^. At present, China still mainly relies on high-pollution, high-emission resource-intensive industries to drive exports. The increase in export volume consumes too much energy and increases CO_2_ emissions, thereby reducing TCE. The ratio of total import and export to GDP is used as a measurement index.
5. Human capital (HUM): The impact of human capital on TCE mainly includes the following two aspects: For one thing, the improvement of human capital level can increase residents’ income^81^, thus changing residents’ consumption demand. Residents’ demand for high-carbon products increases. For meeting the needs of residents, enterprises produce more high-carbon products, which reduces TCE. For another, the improvement of human capital level can improve residents’ awareness of emission reduction and enterprises’ emission reduction technology^82^, thus improving TCE. The ratio of the general college and above population to the total population is used as a measurement index.

#### Data sources and descriptive statistics

This paper selects the panel data of 283 cities in China from 2010 to 2019 as the research sample. The data of CO_2_ emissions are from the CEADs database. The data of fixed asset investment index and GDP deflator are from *China Statistical Yearbook*. Other data are derived from *China Urban Statistical Yearbook*. The descriptive statistics and correlation matrix of variables are shown in Tables 2 and 3 respectively.

**Table 2:** Descriptive statistics of variables.

Variable	Meaning	Calculation	Obs	Mean	Std. dev.	Min	Max
TCE	Total factor carbon emissions efficiency	Global Malmquist index based on SBM model	2830	0.8377	0.1703	0.2084	1.7192
INA	Industrial co-agglomeration	$INA_{i} = \left( {1 - \frac{{\left| {LQ_{im} - LQ_{is} } \right|}}{{LQ_{im} + LQ_{is} }}} \right) + (LQ_{im} + LQ_{is} )$	2830	2.3915	0.5543	0.6112	4.3190
lnGDP	Economic development	Per capita GDP	2830	10.1169	0.5682	8.4646	12.6830
GOV	Government intervention	Government fiscal expenditure/GDP	2830	0.2381	0.2500	0.0438	6.0406
ROAD	Transportation infrastructure level	Road area per capita	2830	0.1299	0.0891	0.0059	1.0837
OPEN	Openness degree	Total import and export/GDP	2830	0.2002	0.3756	0.000035	8.1339
HUM	Human capital	Ordinary undergraduate and above population/total population	2830	1.6993	1.9979	0.0048	12.7642

**Table 3:** Correlation matrix of variables.

	TCE	INA	lnGDP	GOV	ROAD	OPEN	HUM
TCE	1						
INA	0.025* (0.0833)	1					
lnGDP	0.139*** (0.0000)	0.557*** (0.0000)	1				
GOV	− 0.398*** (0.0000)	− 0.289*** (0.0000)	− 0.382*** (0.0000)	1			
ROAD	− 0.120*** (0.0000)	0.262*** (0.0000)	0.450*** (0.0000)	− 0.094*** (0.0000)	1		
OPEN	− 0.046** (0.0154)	0.393*** (0.0000)	0.395*** (0.0000)	− 0.005 (0.798)	0.246*** (0.0000)	1	
HUM	0.049*** (0.0093)	0.353*** (0.0000)	0.445*** (0.0000)	− 0.171*** (0.0000)	0.161*** (0.0000)	0.139*** (0.0000)	1

p value in parentheses.

***p < 0.01, **p < 0.05, *p < 0.1.

Table 3 shows that there is a significant positive correlation between industrial co-agglomeration and TCE. Meanwhile, the absolute values of variables’ correlation coefficients are less than 0.7, which indicates that there is no multicollinearity.

**Figure 4:**
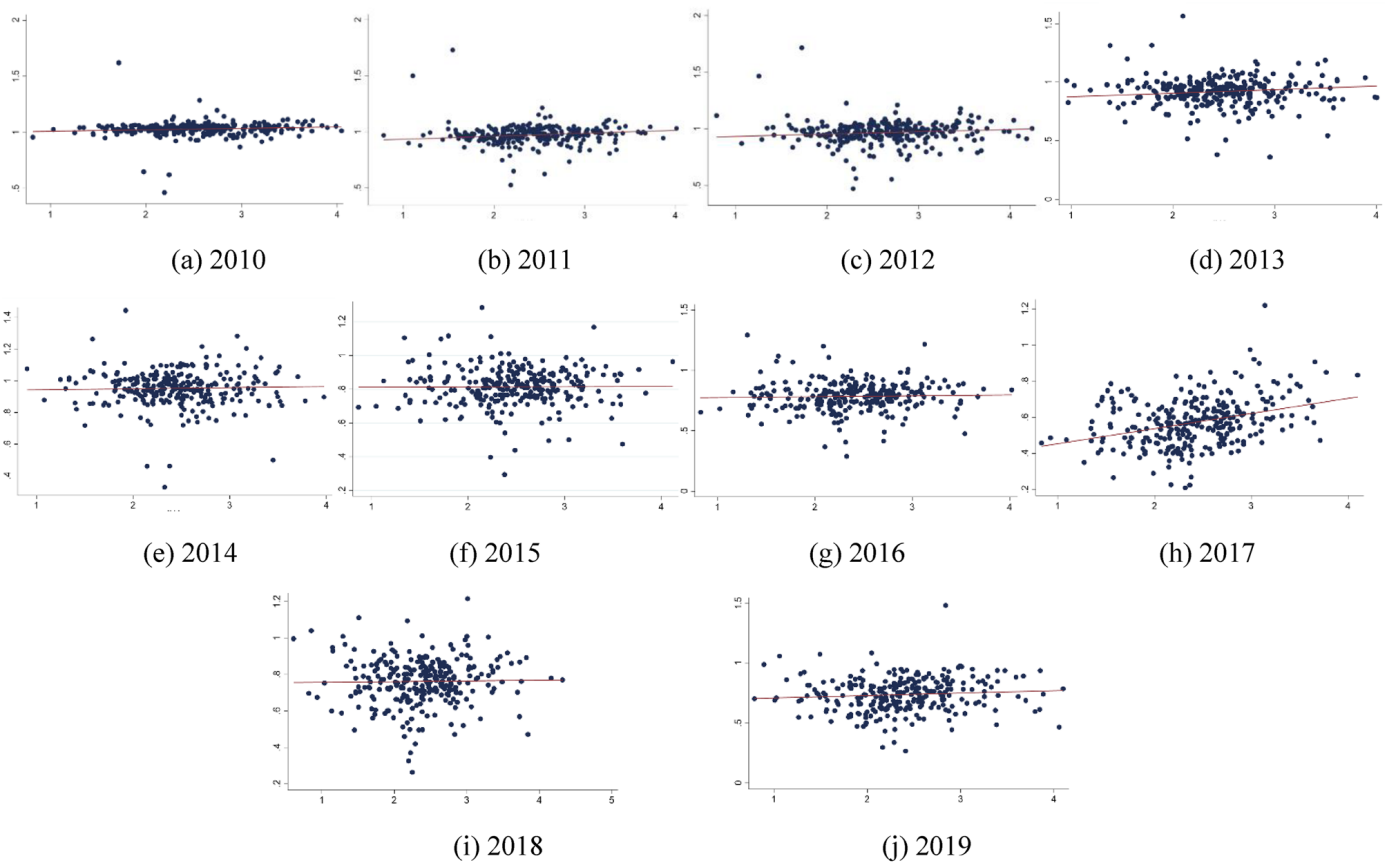
Scatter plot and fitting curve of industrial co-agglomeration and TCE from 2010 to 2019.

The relationship between industrial co-agglomeration and TCE can be further analyzed by distinguishing years. Figure 4 shows the scatter plot and fitting curve of industrial co-agglomeration and TCE from 2010 to 2019. There is a positive correlation between industrial co-agglomeration and TCE from 2010 to 2019. As the level of industrial co-agglomeration increases, TCE gradually increases.

## Result analysis

### Temporal and spatial characteristics of TCE

#### Temporal trend

**Figure 5:**
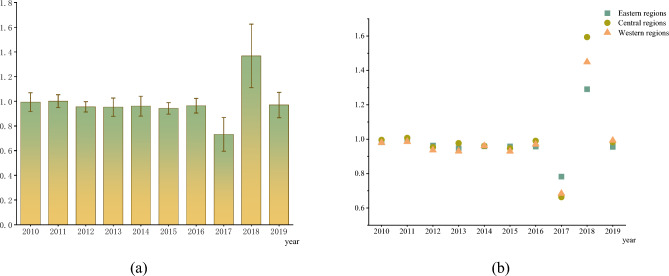
SML mean of China’s TCE from 2010 to 2019.

Figure 5 shows the trend of China’s TCE based on SML index from 2010 to 2019. Overall, the average value of SML index is 0.9836, which indicates that China’s TCE shows a downward trend from 2010 to 2019. Among them, the mean values of SML in 2011 and 2018 are 1.0004 and 1.3679, respectively, indicating that the TCE has increased by 0.04% and 0.3679% in 2011 and 2018, respectively. The mean value of SML in other years is less than 1, indicating that the TCE has decreased in other years. It is worth noting that from 2017 to 2018, the mean value of SML increases significantly. The reasons are as follows: First, the optimization of energy structure. A series of measures are taken to optimize the energy structure, such as controlling the total coal consumption, clean utilization of traditional energy and developing non-fossil energy. In 2018, coal accounts for 59.0% of energy consumption, down 1.4 percentage points from 2017. Natural gas and non-fossil energy account for 7.8% and 14.3% of energy consumption, respectively, which are 0.8 and 0.5 percentage points higher than those in 2017. Second, the improvement of energy utilization efficiency. In 2018, the Ministry of Industry and Information Technology issues 728 industrial energy-saving technology equipment, which not only accelerates the application of energy-saving technology equipment, but also improves energy efficiency. According to statistics, China’s energy consumption per unit of GDP in 2018 is 3.1% lower than that in 2017. Energy utilization efficiency continues to improve, thus greatly improving TCE. Therefore, the mean value of SML in 2018 is significantly higher than that in 2017. From the sub-regional perspective, the SML index of eastern, central and western regions is not much different, which indicates that the TCE of three regions has the same change trend.

#### Spatial distribution

**Figure 6:**
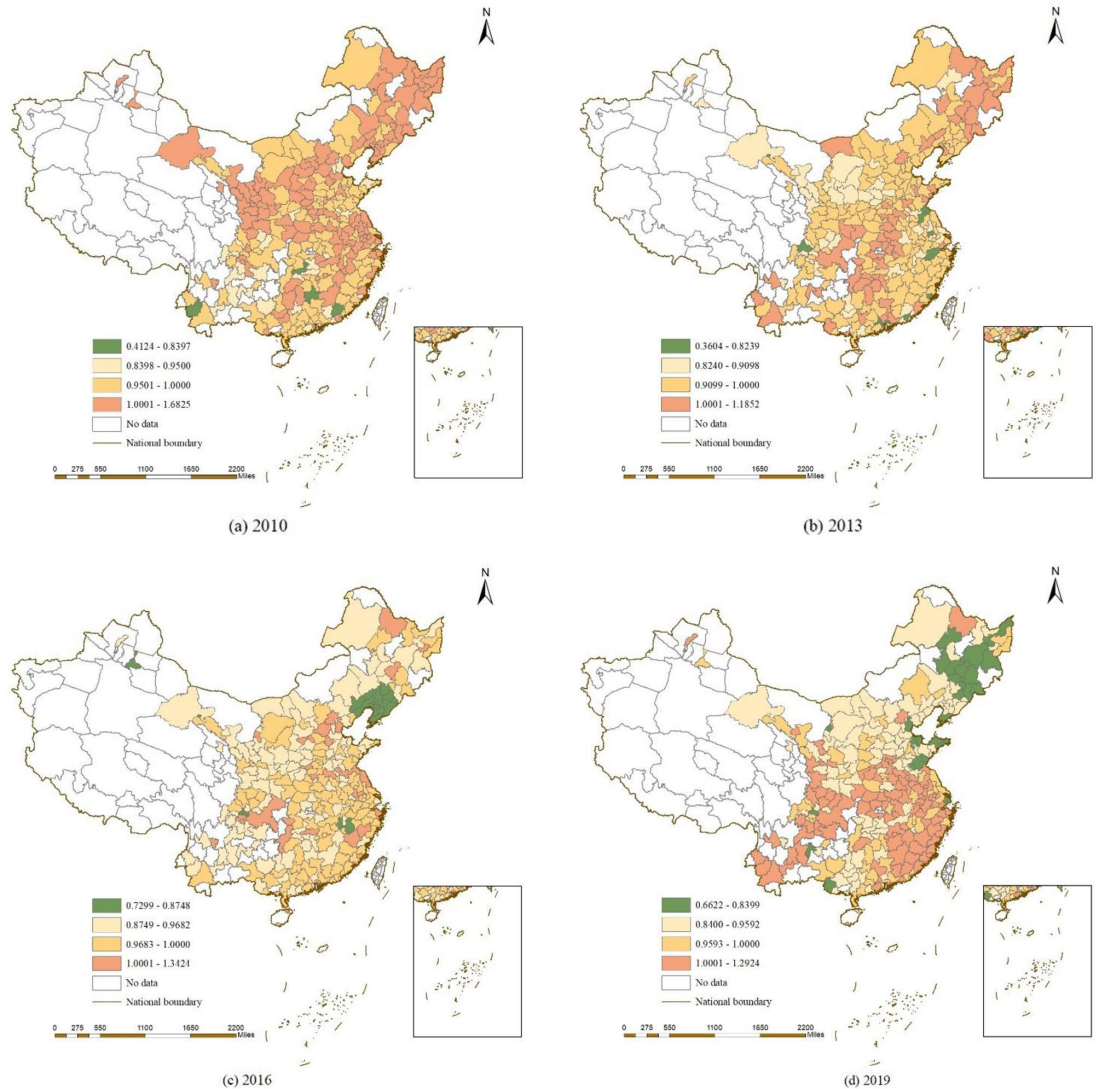
Spatial distribution of TCE in 2009, 2013, 2016 and 2019 (Map created using ArcGIS 10.2, http://www.esri.com/software/arcgis).

Figure 6 shows the spatial distribution characteristics of China’s TCE in 2010, 2013, 2016 and 2019. In 2010, there are 127 cities with improved TCE. In 2013, there are 62 cities with improved TCE. In 2016, there are 34 cities with improved TCE. In 2019, the number of cities with improved TCE increases to 105. Among them, Beijing performs best. its TCE increases in 2010, 2013, 2016 and 2019. In some areas, such as Yantai, Tai’an, Rizhao, Chengdu and Nanning, the TCE decreases in 2010, 2013, 2016 and 2019. It is difficult to achieve CO_2_ reduction targets in these areas.

### Baseline regression analysis

Table 4 reports the estimation results without considering spatial factors. Both F test and LM test pass the 1% significance level, indicating that the fixed effect model and random effect model is superior to mixed OLS. Mixed OLS should not be used. The joint significance test of all annual dummy variables, the Year test, rejects the null hypothesis of “no time effect”. Therefore, in the fixed effect model, the region and time-fixed effect model is the most suitable model. The Hausman test shows that the region and time-fixed effect model should be used in the region and time-fixed effect model and random effect model. Therefore, this paper mainly explains the estimation results of region and time-fixed effect model.

**Table 4:** Baseline regression results.

	OLS	Regional fixed effects	Time fixed effects	Regional and time-fixed effects	Random effects
INA	0.0528*** (0.0074)	0.0757*** (0.0150)	0.0284*** (0.0048)	0.0170** (0.0069)	0.0458*** (0.0121)
lnGDP	0.0569*** (0.0098)	0.388*** (0.0213)	0.0455*** (0.0055)	0.249*** (0.0136)	0.215*** (0.0144)
GOV	− 0.419*** (0.0169)	− 0.485*** (0.0128)	− 0.0709*** (0.0112)	− 0.211*** (0.0095)	− 0.479*** (0.0133)
ROAD	− 0.486*** (0.0481)	− 0.951*** (0.0734)	− 0.162*** (0.0273)	− 0.189*** (0.0472)	− 0.969*** (0.0628)
OPEN	0.0004 (0.0116)	− 0.0337*** (0.0127)	− 0.0141** (0.0065)	− 0.0439*** (0.0079)	− 0.0329*** (0.0123)
HUM	0.0019 (0.0021)	− 0.104*** (0.0080)	0.0040*** (0.0012)	− 0.0164*** (0.0052)	− 0.0286*** (0.0039)
Constant	4.157*** (0.0932)	1.134*** (0.218)	0.479*** (0.0529)	2.227*** (0.138)	2.757*** (0.138)
*N*	2830	2830	2830	2830	2830
R^2^	0.241	0.520	0.070	0.821	0.494
F test		12.00***	29.61***	24.18***	
LM test					1747.05***
Year test			15.33***		
Hausman test		424.26***	622.51***	863.97***	

Standard errors in parentheses.

***p < 0.01, **p < 0.05, *p < 0.1.

The regression coefficient of industrial co-agglomeration is significantly positive (0.0170, 0.01 < p < 0.05), which indicates that industrial co-agglomeration can improve TCE. Regarding control variables, economic development has a significant positive impact on TCE. Government intervention, transportation infrastructure level, openness and human capital have a significant negative impact on TCE.

Since panel data are spatially correlated, ignoring spatial correlation may make the estimation results inaccurate^83^. Therefore, the spatial econometric model is further used.

### Spatial correlation test

#### Global Moran’s Index

Table 5 shows the global Moran’s Index of TCE and industrial co-agglomeration from 2010 to 2019. From 2011 to 2019, the global Moran’s Index of TCE and industrial co-agglomeration is significantly positive, which indicates that both TCE and industrial co-agglomeration have a significant positive spatial correlation.

**Table 5:** Global Moran’s I.

Year	2010	2011	2012	2013	2014	2015	2016	2017	2018	2019
TCE	0.041***	0.049***	0.057***	0.081***	0.065***	0.061***	0.039***	0.026***	0.054***	0.049***
INA	0.075***	0.077***	0.086***	0.086***	0.083***	0.084***	0.090***	0.093***	0.092***	0.115***

***p < 0.01, **p < 0.05, *p < 0.1.

#### Local Moran’s Index

**Figure 7:**
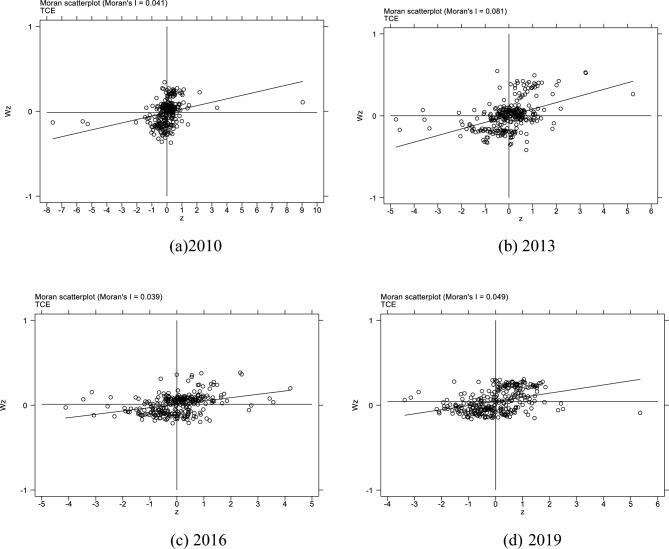
Moran scatter plot of TCE.

**Figure 8:**
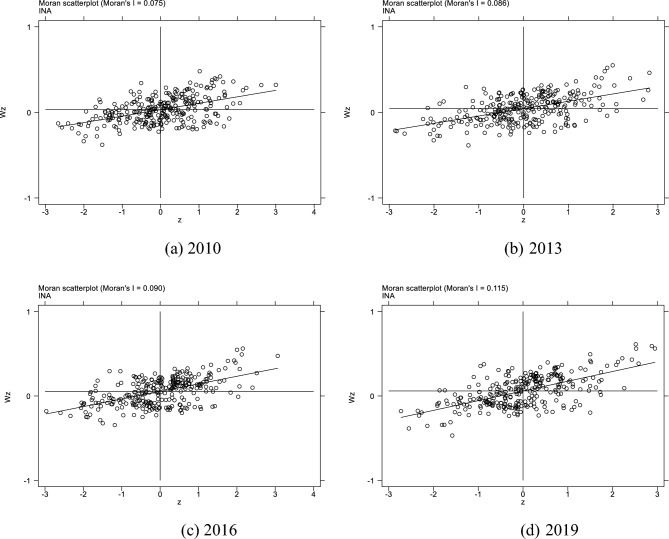
Moran scatter plot of industrial co-agglomeration.

In order to further reflect the spatial agglomeration characteristics of TCE and industrial co-agglomeration, this paper draws Moran scatter plots. Figures 7 and 8 show the local Moran scatter plots of TCE and industrial co-agglomeration in 2010, 2013, 2016 and 2019 respectively. Most cities are located in the first and third quadrants, indicating that both TCE and industrial co-agglomeration have significant positive spatial agglomeration characteristics.

### Empirical results of spatial econometric model

This paper selects the spatial econometric model to further analyze the impact of industrial co-agglomeration on TCE. The estimation results are shown in Table 6.

**Table 6:** Estimation results of spatial econometric model.

	SAR FE	SAR RE	SEM FE	SEM RE	SDM FE	SDM RE
INA	0.0205*** (0.0063)	0.0304*** (0.0059)	0.0211*** (0.0064)	0.0211*** (0.0064)	0.0255*** (0.0064)	0.0307*** (0.0061)
lnGDP	0.178*** (0.0091)	0.126*** (0.0082)	0.196*** (0.0099)	0.196*** (0.0099)	0.189*** (0.0107)	0.125*** (0.0095)
GOV	− 0.119*** (0.0064)	− 0.0972*** (0.0063)	− 0.120*** (0.0064)	− 0.121*** (0.0064)	− 0.118*** (0.0064)	− 0.113*** (0.0068)
ROAD	− 0.103*** (0.0317)	− 0.181*** (0.0308)	− 0.0936*** (0.0315)	− 0.0940*** (0.0315)	− 0.105*** (0.0315)	− 0.143*** (0.0310)
OPEN	− 0.0295*** (0.0053)	− 0.0265*** (0.0054)	− 0.0288*** (0.0053)	− 0.0287*** (0.0053)	− 0.0293*** (0.0053)	− 0.0308*** (0.0055)
HUM	− 0.0111*** (0.0035)	− 0.0093*** (0.0024)	− 0.0104*** (0.0035)	− 0.0105*** (0.0035)	− 0.0096*** (0.0034)	− 0.0077*** (0.0024)
W*TCE	0.877*** (0.0365)	0.873*** (0.0112)			0.867*** (0.0403)	0.832*** (0.0396)
W*INA					0.304*** (0.0792)	0.0275 (0.0325)
*N*	2830	2830	2830	2830	2830	2830
R^2^	0.414	0.472	0.070	0.070	0.498	0.510
Hausman test	45.15***	45.15***	52.36***	52.36***	66.85***	66.85***
Wald test spatial lag					51.69***	52.67***
Wald test spatial error					57.65***	28.94***
LR test spatial lag					51.28***	52.38***
LR test spatial error					55.88***	49.25***

Standard errors in parentheses.

***p < 0.01, **p < 0.05, *p < 0.1.

The spatial lag coefficient of TCE is significantly positive, indicating that TCE has a positive spatial spillover effect. China’s promotion competition mechanism has increased the promotion pressure of local government officials, which encourages low-TCE areas to actively learn from the low-carbon technology and management experience of surrounding high-TCE areas. TCE in the region is improved. Meanwhile, using the spatial econometric models is necessary. The Wald and LR test results shows that SDM model cannot be simplified into SAR model and SEM model. Hausman test results show that the fixed effects should be selected in the fixed effects and random effects. Therefore, this paper selects the SDM model with fixed time and region to explain the estimation results.

Regarding industrial co-agglomeration, the estimated coefficient of industrial co-agglomeration is 0.0255, and it passes the 1% significant level, which conforms to the hypothesis 1. The estimated coefficient of the spatial lag term of industrial co-agglomeration is 0.304, and it also passes the 1% significance level, which is in line with the hypothesis 3. Industrial co-agglomeration has a positive spatial spillover effect on TCE.

Regarding control variables, economic development has a positive impact on TCE. At present, China’s economy is at a high level. The quality of economic growth is more important than that of economic growth. The government takes more stringent environmental protection measures to reduce CO_2_ emissions. For promoting economic growth, the government invests massive money in the field of high economic returns, which squeezes investment in the field of environmental protection and reduces TCE. Similarly, the construction of transportation infrastructure requires a large amount of fossil energy, which produces a large amount of CO_2_ and reduces TCE. In addition, the degree of openness and human capital level also have a significant negative impact on TCE.

In order to fully reflect the marginal effect of explanatory variables on explained variables, this paper gives the direct effect, indirect effect and total effect of each variable in Table 7. The direct effect, indirect effect and total effect of industrial co-agglomeration are significantly positive, which is similar to the estimation results in Table 6. It is worth noting that the estimation coefficient of indirect effect of industrial co-agglomeration is greater than that of direct effect, which indicates that the industrial co-agglomeration of surrounding regions has a greater impact on TCE of the region. The technology spillover effect and learning effect generated by industrial co-agglomeration are mainly reflected between regions, not within regions. As for technology spillover effect, the continuous improvement of transportation infrastructure enhances the mobility of talents, knowledge and technology. The knowledge and technology of the surrounding areas gradually spread to the region. As for learning effect, if the positive externalities generated by industrial co-agglomeration in the surrounding areas are higher than those in the region, the region will take the surrounding areas as an example and actively introduce relevant industrial policies due to competitive pressure. The positive externality of industrial co-agglomeration in the region is enhanced. With the increase of inter-regional competition pressure, the learning effect is increasing, which makes the spatial spillover effect of industrial co-agglomeration on TCE greater than direct effect.

**Table 7:** Direct effect, indirect effect and total effect.

	Direct effect	Indirect effect	Total effect
INA	0.0162* (0.0085)	0.4985** (0.2509)	0.5147** (0.2595)
lnGDP	0.188*** (0.0097)	0.0481 (0.501)	0.236 (0.499)
GOV	− 0.125*** (0.0217)	− 2.145 (5.902)	− 2.270 (5.923)
ROAD	− 0.153 (0.124)	− 13.29 (33.94)	− 13.44 (34.06)
OPEN	− 0.0304*** (0.0092)	− 0.295 (2.242)	− 0.325 (2.250)
HUM	− 0.0150 (0.0124)	− 1.550 (3.388)	− 1.565 (3.400)

Standard errors in parentheses.

***p < 0.01, **p < 0.05, *p < 0.1.

### Transmission mechanism analysis

For testing the transmission mechanism, this paper introduces the intermediary variable of technological innovation and constructs the intermediary effect model. The specific formula is as follows:

10 $$\begin{aligned} TCE_{it} & = \theta_{0} \sum\limits_{j} {W_{ij} } TCE_{it} + \theta_{1} INA_{it} + \theta_{2} \ln GDP_{it} + \theta_{3} GOV_{it} + \theta_{4} ROAD_{it} \\ & \quad + \theta_{5} OPEN_{it} + \theta_{6} HUM_{it} + \rho_{0} \sum\limits_{j} {Wij} INA_{it} + \rho_{1} \sum\limits_{j} {Wij} \ln GDP_{it} \\ & \quad + \rho_{2} \sum\limits_{j} {Wij} GOV_{it} + \rho_{3} \sum\limits_{j} {Wij} ROAD_{it} + \rho_{4} \sum\limits_{j} {Wij} OPEN_{it} \\ & \quad + \rho_{5} \sum\limits_{j} {W_{ij} } HUM_{it} + \mu_{i} + \lambda_{t} + \varepsilon_{it} \\ \end{aligned}$$

11 $$\begin{aligned} INNOV_{it} & = \chi_{0} \sum\limits_{j} {W_{ij} } INNOV_{it} + \chi_{1} INA_{it} + \chi_{2} \ln GDP_{it} + \chi_{3} GOV_{it} + \chi_{4} ROAD_{it} \\ \, & \quad + \chi_{5} OPEN_{it} + \chi_{6} HUM_{it} + \eta_{0} \sum\limits_{j} {W_{ij} } INA_{it} + \eta_{1} \sum\limits_{j} {W_{ij} } \ln GDP_{it} \\ & \quad + \eta_{2} \sum\limits_{j} {W_{ij} } GOV_{it} + \eta_{3} \sum\limits_{j} {W_{ij} } ROAD_{it} + \eta_{4} \sum\limits_{j} {W_{ij} } OPEN_{it} \\ & \quad { + }\eta_{5} \sum\limits_{j} {W_{ij} } HUM_{it} + \mu_{i} + \lambda_{t} + \varepsilon_{it} \\ \end{aligned}$$

12 $$\begin{aligned} TCE_{it} & = \varphi 0\sum\limits_{j} {W_{ij} } TCE_{it} + \varphi_{1} INA_{it} + \varphi_{2} INNOV_{it} + \varphi_{3} \ln GDP_{it} + \varphi_{4} GOV_{it} \\ & \quad + \varphi_{5} ROAD_{it} + \varphi_{6} OPEN_{it} + \varphi_{7} HUM_{it} + \omega_{0} \sum\limits_{j} {W_{ij} } INA_{it} + \omega_{1} \sum\limits_{j} {INNOV_{it} } \\ & \quad + \omega_{2} \sum\limits_{j} {W_{ij} } \ln GDP_{it} + \omega_{3} \sum\limits_{j} {W_{ij} } GOV_{it} + \omega_{4} \sum\limits_{j} {W_{ij} } ROAD_{it} + \omega_{5} \sum\limits_{j} {W_{ij} } OPEN_{it} \\ & \quad + \omega_{6} \sum\limits_{j} {W_{ij} } HUM_{it} + \mu_{i} + \lambda_{t} + \varepsilon_{it} \\ \end{aligned}$$

**Figure 9:**
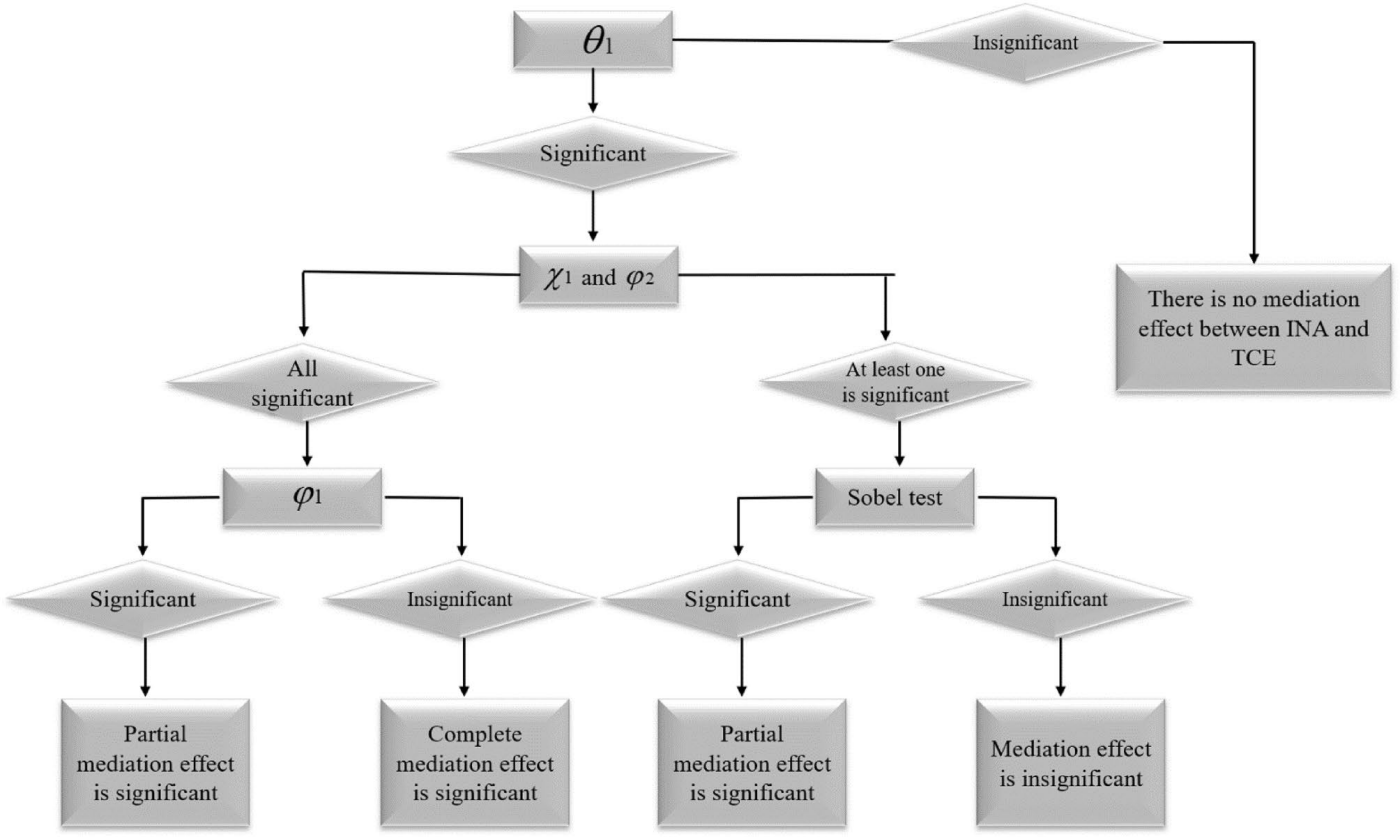
Mediation effect test process.

Among them, *INNOV* is the mediating variable of technological innovation. This paper selects the number of patent applications per capita as its measurement index. According to the principle of mediating effect, the total effect, direct effect and mediating effect should satisfy $\theta_{1} = \varphi_{1} + \chi_{1} \times \varphi_{2}$. Figure 9 shows the specific process of mediating effect test.

The estimation results are shown in Table 8. Industrial co-agglomeration can improve the TCE through technological innovation. Moreover, technological innovation plays a partial mediating effect in the process of industrial co-agglomeration affecting TCE. The mediating effect accounts for 21.15% of the total effect.

**Table 8:** Transmission mechanism test.

	INNOV	TCE	TCE
INA	0.1620*** (0.0045)	0.0201*** (0.0064)	0.0255*** (0.0064)
INNOV		0.0333*** (0.0058)	
W*TCE		0.867*** (0.0403)	0.867*** (0.0403)
W*INA	0.988*** (0.235)	0.282*** (0.0797)	0.304*** (0.0792)
W*INNOV	0.8508*** (0.0441)	− 0.0176 (0.0446)	
Control variables	Yes	Yes	Yes
*N*	2830	2830	2830
R^2^	0.021	0.491	0.498

Standard errors in parentheses.

***p < 0.01, **p < 0.05, *p < 0.1.

### Robustness test

#### Replace the explained variable

This paper takes CO_2_ emissions per unit of GDP as a measure of TCE. The estimation results are shown in Column (1) of Table 9. Industrial co-agglomeration can reduce CO_2_ emissions per unit of GDP, which proves the robustness of above estimation results.

**Table 9:** Robustness test.

	(1)	(2)	(3)
INA	− 0.0062* (0.0037)	0.124*** (0.0006)	0.0158** (0.0063)
L.TCE		0.215*** (0.0214)	
W*TCE	0.853*** (0.0442)	0.594*** (0.0075)	0.898*** (0.0313)
W*INA	− 0.0802* (0.0460)	0.511*** (0.0052)	0.224*** (0.0797)
Control variables	Yes	Yes	Yes
AR(1)		0.000	
AR(2)		0.500	
Hansen test		0.175	
*N*	2830	2547	2790

Standard errors in parentheses.

***p < 0.01, ** p < 0.05, * p < 0.1.

#### Replace estimation method

In order to solve the possible endogenous problems, this paper uses a spatial system GMM model to re-estimate the impact of industrial co-agglomeration on TCE. Columns (2) of Table 9 shows that industrial co-agglomeration still has a positive impact on TCE.

#### Replace sample

The economic development of Beijing, Tianjin, Shanghai and Chongqing has a big gap with other cities. Therefore, this paper excludes four municipalities from the sample. The estimated results are shown in Column (3) of Table 9. The regression coefficient of industrial co-agglomeration is still significantly positive.

### Heterogeneity analysis

#### Geographical location

The economic development of each region has significant differences, which makes the impact of industrial co-agglomeration on TCE in different regions may be different. Dividing 283 cities in China into eastern, central and western regions, this paper deeply discusses the heterogeneous impact of industrial co-agglomeration on TCE in different regions.

The estimated results are shown in Table 10. Among them, the impact of industrial co-agglomeration on TCE in the eastern and central regions is significantly positive, and the promotion effect in the eastern region is greater than that in the central region. The impact of industrial co-agglomeration on TCE in the western region is not significant. Relying on the advantages of previous policies and geographical location, the eastern region has accumulated massive industrial resources. The talent reserve and industrial level are higher than the national average, which lays a good foundation for the coordinated agglomeration of industries. The accumulation of capital, labor and other production resources can promote the positive externalities of industrial co-agglomeration, thus improving TCE. Compared with the eastern region, the economic development and industrial structure in the central region is lower. Industrial co-agglomeration may bring about problems such as low production efficiency and environmental pollution, thus reducing the positive externalities of industrial co-agglomeration. Limited by geographical conditions, economic development, industrial structure and human capital in the western region are low, which makes it difficult for industrial co-agglomeration to exert positive externalities.

**Table 10:** Heterogeneity.

	Eastern regions	Central regions	Western regions	Resource-based cities	Non-resource-based cities
INA	0.0402*** (0.0135)	0.0196* (0.0109)	0.0138 (0.0130)	0.0157* (0.0084)	0.0381*** (0.0092)
W*TCE	0.667*** (0.0767)	0.664*** (0.0829)	− 0.333 (0.206)	0.747*** (0.0707)	0.722*** (0.0766)
W*INA	0.242*** (0.0644)	0.441*** (0.107)	− 0.295*** (0.100)	− 0.0143 (0.0795)	0.532*** (0.0789)
Control variables	Yes	Yes	Yes	Yes	Yes
*N*	1000	1000	830	1140	1690
R^2^	0.266	0.221	0.353	0.528	0.239

Standard errors in parentheses.

***p < 0.01, ** p < 0.05, * p < 0.1.

#### Resource endowment

Different cities have different natural resource endowments, which makes the impact of industrial co-agglomeration on TCE different. According to the *National Sustainable Development Plan for Resource-based Cities* (*2013–2020*) issued by the State Council, this paper divides 283 cities in China into 114 resource-based cities and 169 non-resource-based cities (Appendix A).

The estimated results are shown in Table 10. The promotion effect of non-resource-based cities is greater than that of resource-based cities. The leading industries of resource-based cities are heavy and single, which has a crowding-out effect on the development of other industries. The crowding-out effect weakens the positive impact of industrial co-agglomeration on TCE. Zhang et al.^14^, Guo et al.^84^, Li et al.^85^ all believed that industrial agglomeration might have a crowding-out effect on other industries, thus exacerbating environmental pollution. Liu et al.^54^ found that industrial agglomeration level could hinder the development of other industries and the entry of enterprises, which reduced carbon productivity. Therefore, the promotion effect of industrial co-agglomeration on TCE in resource-based cities less than that of non-resource-based cities.

According to Cleary^86^, this paper uses the Fisher’s Permutation test to test the difference between coefficient arrays. The sampling frequency is 1000. Test results are shown in Table 11. The p value of the difference test between coefficient groups is less than 0.1, which indicates that the impact of industrial co-agglomeration on TCE is significantly different in cities with different geographical locations and different resource endowments.

**Table 11:** Test of the difference between coefficient arrays.

	Coefficient	p value
Eastern regions versus Central regions	0.060	0.000
Eastern regions versus Western regions	0.045	0.003
Central regions versus Western regions	0.104	0.000
Resource-based cities versus Non-resource-based cities	0.079	0.012

## Discussion

Industrial co-agglomeration has a positive impact on TCE. For one thing, industrial co-agglomeration can expand the supply and demand of intermediate goods and reduce the transaction costs of intermediate goods. Meanwhile, industrial co-agglomeration can also attract the inflow of talents and reduce the cost of information collection and talent recruitment. Cost reduction can increase the economic benefits of enterprises, which enables enterprises to carry out technology research and development activities and improve TCE. For another, industrial co-agglomeration can accelerate the transfer of production factors from low-efficiency enterprises to high-efficiency enterprises, which improves the efficiency of resource allocation and thus improves TCE. In the *Implementation Opinions on Promoting the Deep Integration of Advanced Manufacturing Industry and Modern Service Industry* issued by the Chinese government, it is pointed out that the integration of advanced manufacturing industry and modern productive service industry is an important way to achieve sustainable economic development. In order to respond positively to the call, Liaoning Province has issued relevant support policies to reward enterprises with the title of service-oriented manufacturing demonstration enterprises. By the end of 2022, Liaoning Province has identified 284 provincial service-oriented manufacturing demonstration enterprises. Among them, there are 41 national service-oriented manufacturing demonstration enterprises, ranking fifth in China.

Industrial co-agglomeration can improve TCE through technological innovation. Industrial co-agglomeration can promote the spillover of knowledge and technology in the industry, which is conducive to the development of production and emission reduction technology. Meanwhile, industrial co-agglomeration can realize the sharing of resources such as labor, education and infrastructure, reduce the cost of innovation, and promote the development of technological innovation activities. Technological innovation can not only improve the utilization efficiency of factors, but also improve the energy structure, thereby reducing CO_2_ emissions from the source. According to the National Energy Statistics Bureau, clean energy accounts for 42.4% of total installed capacity in 2020, an increase of 14.6% over 2012. This is mainly due to the progress of new energy technology and material technology.

Industrial co-agglomeration has a positive spatial spillover effect on TCE. Firstly, industrial co-agglomeration can promote the diffusion of knowledge and technology to the surrounding areas, which makes the TCE of surrounding areas increase. Secondly, under the pressure of political promotion, the surrounding areas can learn from the advanced technology and management experience of the area, which makes economic growth while reducing CO_2_ emissions. Thirdly, industrial co-agglomeration can promote regional specialized production. Technology-intensive industries are mainly concentrated in areas with high productivity levels, while resource and labor-intensive industries are gradually transferred to areas with low productivity levels. Each region realizes specialized division of labor on the basis of the original industry. Complementary advantages are realized, which improves TCE. At present, Shanghai’s leading industries are gradually shifting to services such as finance, business services and electronic information technology, which greatly strengthens Shanghai’s core economic influence. Seizing the development opportunity of Shanghai 's industrial transformation, Jiangsu, Zhejiang and Anhui actively undertake the transferred industries. Based on their own development, these cities continue to improve the degree of industrial specialization, which promotes the overall development.

## Conclusions and policy recommendations

This paper uses the global Malmquist index based on SBM to measure the TCE of 283 cities in China from 2011 to 2019. On this basis, this paper uses spatial econometric model and mediating effect model to empirically test the impact of industrial co-agglomeration on TCE and its mechanism. Meanwhile, according to geographical location, the total sample is divided into three sub-samples: Eastern, central and western. According to resource endowment, 283 cities are divided into resource-based cities and non-resource-based cities. This paper further analyzes the heterogeneous impact of industrial co-agglomeration on TCE in cities with different geographical locations and resource endowments. The conclusions are as follows: (1) Industrial co-agglomeration can improve TCE. For every 1 unit increase in the industrial co-agglomeration index, TCE increases by 0.0255 units. (2) Industrial co-agglomeration has a positive spatial spillover effect on TCE, which mainly comes from technology spillover, demonstration effect and collaboration effect. (3) Technological innovation plays a partial mediating effect in the process of industrial co-agglomeration improving TCE. The mediating effect accounted for 21.15% of the total effect. (4) Heterogeneity analysis shows that industrial co-agglomeration in different regions has heterogeneous effects on TCE. Among them, the industrial co-agglomeration in the eastern region has the greatest promoting effect on TCE, followed by the central region, while the industrial co-agglomeration in the western region has no significant effect on TCE. The industrial co-agglomeration of cities with different resource endowments has different effects on TCE. The promoting effect of industrial co-agglomeration on TCE in non-resource-based cities is greater than that in resource-based cities.

According to the conclusion, this paper puts forward the following policy recommendations:

1. Adhere to the industrial coordinated development strategy, strengthen the integration of manufacturing and producer services. Local governments should adhere to the two-wheel drive strategy of “manufacturing + service” to stimulate the cost effect, resource allocation effect, innovation effect and spatial spillover effect brought by industrial co-agglomeration. While focusing on the coordinated development of industries, all regions should actively develop environmentally friendly industries. Through using personnel training, innovation incentives and other ways to improve the proportion of environmentally friendly industries in the productive service industry.
2. Improve technology and talent policy, promote technological innovation. The core driving force of industrial co-agglomeration to improve TCE is technological innovation. The key to technological innovation lies in the development of science and technology and introduction of talents. Therefore, local governments should increase investment in science and technology and talents, and give certain financial incentives to high-tech industries. Talent and low-carbon technology introduction policies should also be actively introduced. Meanwhile, the protection of intellectual property rights should be increased to improve the innovation enthusiasm of enterprises.
3. Develop industrial policies according to local conditions, achieve rationalization of industrial layout. At present, the degree of integration of manufacturing and service industries in some regions is not high, which makes TCE under industrial co-agglomeration still have a lot of room for improvement. Therefore, each region should create an industrial layout based on the actual situation of its own manufacturing and producer services. Meanwhile, when formulating industrial policies, regions should fully consider the coordination of inter-regional industrial policies. The linkage development of industries in various regions should be accelerated to give full play to the synergistic agglomeration effect of industries.

## Supplementary Information

Supplementary Information.

## Data Availability

The data that support the findings of this study are available from [https://data.cnki.net/] but restrictions apply to the availability of these data, which are used under license for the current study, and so are not publicly available. Data are however available from the authors upon reasonable request and with permission of [https://data.cnki.net/]. Those wishing to request data from this study can contact Jia Liu.
