# Supplementary material for: The impact of industrial collaborative agglomeration on total factor carbon emission efficiency in China
Source: Sci Rep. 2023 Jul 31;13:12347. doi: 10.1038/s41598-023-39631-3 (PMC10390566; doi:10.1038/s41598-023-39631-3)
Supplement: Supplementary file 1 — Supplementary Information. [file 41598_2023_39631_MOESM1_ESM.docx]

**Appendix A**

Resource-based cities and non-resource-based cities

| Province | Resource-based city | Non-resource-based city |
| --- | --- | --- |
|  |  | Beijing |
|  |  | Tianjin |
| Hebei | Tangshan、Handan、Xingtai、Zhangjiakou、Chengde | Shijiazhuang、Qinhuangshan、Baoding、Cangzhou、Langfang、Hengshui |
| Shanxi | Datong、Yangquan、Changzhi、Jincheng、Shuozhou、Jinzhong、Yuncheng、Qizhou、Linfen、Luliang | Taiyuan |
| Inner Mongolia | Baotou、Wuhai、Chifeng、Ordos、Hulunbuir | Hohhot、Tongliao、Bayannur、Ulanqab |
| Liaoning | Anshan、Fushun、Benxi、Fuxin、Panjin、Huludao | Shenyang、Dalian、Dandong、Jinzhou、Yingkou、Liaoyang、Tieling、Chaoyang |
| Jilin | Jilin、Liaoyuan、Tonghua、Baishan、Songyuan | Changchun、Siping、Baicheng |
| Heilongjiang | Jixi、Hegang、Shuangyashan、Daqing、Yichun、Qitaihe、Mudanjiang、Heihe | Harbin、Qiqihar、Jiamusi、Suihua |
|  |  | Shanghai |
| Jiangsu | Xuzhou、Suqian | Nanjing、Wuxi、Changzhou、Suzhou、Nantong、Lianyungang、Huaian、Yancheng、Yangzhou、Zhenjiang、Taizhou |
| Zhejiang | Huzhou | Hangzhou、Ningbo、Wenzhou、Jiaxing、Shaoxing、Jinhua、Quzhou、Zhoushan、Taizhou、Lishui |
| Anhui | Huainan、Maanshan、Huaibei、Tongling、Chuzhou、Suzhou、Bozhou、Chizhou、Xuancheng | Hefei、Wuhu、Bengbu、Anqing、Huangshan、Fuyang、Luan |
| Fujian | Sanming、Nanping、Longyan | Fuzhou、Xiamen、Putian、Quanzhou、Zhangzhou、Ningde |
| Jiangxi | Jingdezhen、Pingxiang、Xinyu、Ganzhou、Yichun | Nanchang、Jiujiang、Yingtan、Jian、Fuzhou、Shangrao |
| Shandong | Zibo、Zaozhuang、Dongying、Jining、Taian、Linyi | Jinnan、Qingdao、Yantai、Weifang、Weihai、Rizhao、Dezhou、Liaocheng、Binzhou、Heze |
| Henan | Luoyan、Pingdingshan、Hebi、Jiaozuo、Puyang、Sanmenxia、Nanyang | Zhengzhou、Kaifeng、Anyang、Xinxiang、Xuchang、Luohe、Shangqiu、Xinyang、Zhoukou、Zhumadian |
| Hubei | Huangshi、Ezhou | Wuhan、Shiyan、Yichang、Xiangyang、Jingmen、Xiaogan、Jingzhou、Huanggang、Xianning、Suizhou |
| Hunan | Hengyang、Shaoyang、Benzhou、Loudi | Changsha、Zhuzhou、Xiangtan、岳Yueyang、Changde、Zhangjiajie、Yiyang、Yongzhou、Huaihua |
| Guangdong | Shaoguan、Yunfu | Guangzhou、Shenzhen、Zhuhai、Shantou、Foushan、Jiangmen、Zhenjiang、Maoming、Zhaoqing、Huizhou、Meizhou、Shanwei、Heyuan、Yangjiang、Qingyuan、Dongguan、Zhongshan、Chaozhou、Jieyang |
| Guangxi | Hezhou、Hechi | Nanning、Liuzhou、Wuzhou、Beihai、Fangchengang、Qinzhou、Guigang、Yulin、Laibin、Chongzuo |
| Hainan |  | Haikou、Sanya |
|  |  | Chongqing |
| Sichuan | Zigong、Panzhihua、Luzhou、Guangyuan、Nanchong、Guangan、Dazhou、Yaan | Chengdu、Deyang、Mianyang、Suining、Neijiang、Leshan、Meishan、Yibin、Bazhong、Ziyang |
| Guizhou | Liupanshui、Anshun | Guiyang、Zunyi |
| Yunnan | Qujing、Baoshan、Zhaotong、Lijiang、Puer、Lincang | Kunming、Yuxi |
| Shanxi | Tongchuan、Baoji、Xianyang、Weinan、Yanan、Yulin | Xian、Hanzhong、Ankang、Shangluo |
| Gansu | Jinchang、Baiyin、Wuwei、Zhangye、Pingliang、Qingyang、Longnan | Lanzhou、Jiayuguan、Tianshui、Jiuquan、Dingxi |
| Qinghai |  | Xining |
| Ningxia | Shizuishan | Wuzhong、Guyuan、Zhongwei |
| Xinjiang | Karamay | Urumqi |
